# Xpert MTB/RIF assay for the differential diagnosis between sarcoidosis and tuberculosis intrathoracic lymphadenopathy

**DOI:** 10.1186/s12879-023-08734-7

**Published:** 2023-10-25

**Authors:** Xian He, Yuan Zhang, Ying Zhou, Li Li, Qiuhong Li

**Affiliations:** 1 Department of Respiratory and Critical Care Medicine, Shanghai Pulmonary Hospital, School of Medicine, Tongji University, 507 Zheng Min Road, Shanghai, 200433 China; 2 Department of Respiratory Disease, Baoshan District Hospital of Integrated Traditional Chinese and Western Medicine, Shanghai, China

**Keywords:** Sarcoidosis, Tuberculosis, Intrathoracic lymphadenopathy, Xpert MTB/RIF, QuantiFERON-TB gold

## Abstract

**Background:**

The aim of this study was to evaluate the role of Xpert MTB/RIF assay in the detection of *Mycobacterium tuberculosis* for differentiating tuberculosis intrathoracic lymphadenopathy from sarcoidosis intrathoracic lymphadenopathy.

**Methods:**

The patients who were suspected to having sarcoidosis or tuberculosis intrathoracic lymphadenopathy at the Shanghai Pulmonary Hospital between October 1, 2020 and June 30, 2021 were retrospectively evaluated in this study. All patients underwent endobronchial ultrasound-guided transbronchial needle aspiration (EBUS-TBNA) and Xpert analysis. Differences in clinical and radiological features were recorded. The diagnostic performances of EBUS-TBNA Xpert, acid-fast bacilli, culture, and peripheral blood QuantiFERON-TB Gold (QFT) for differentiating sarcoidosis from tuberculosis intrathoracic lymphadenopathy were analyzed.

**Results:**

A total of 119 patients were included in this analysis. Of those, 83 patients were finally diagnosed with sarcoidosis (N = 50) and tuberculosis (N = 33) intrathoracic lymphadenopathy. Young individuals were more likely to have tuberculosis versus sarcoidosis intrathoracic lymphadenopathy (P = 0.006). Markers of inflammation, including fever, leukocytes, and serum ferritin levels, were significantly higher in tuberculosis versus sarcoidosis intrathoracic lymphadenopathy (P < 0.01). Bilateral lung involvement and symmetry intrathoracic lymphadenopathy were more common in sarcoidosis intrathoracic lymphadenopathy (P < 0.01). In addition, the longest diameter of intrathoracic lymphadenopathy (in cm) was significantly larger in sarcoidosis intrathoracic lymphadenopathy (P = 0.001). However, the largest diameter of lung lesions was significantly shorter (P = 0.005). The sensitivity and specificity values of Xpert and QFT for differentiating these two diseases were 69.70% and 100%, and 96.43% and 91.84%, respectively.

**Conclusion:**

Xpert MTB/RIF is recommended for the diagnosis of tuberculosis intrathoracic lymphadenopathy using EBUS-TBNA samples. A negative QFT suggests the exclusion of the diagnosis of tuberculosis intrathoracic lymphadenopathy.

**Supplementary Information:**

The online version contains supplementary material available at 10.1186/s12879-023-08734-7.

## Introduction

Tuberculosis (TB), is a major public health problem worldwide, particularly in China. This common chronic infectious disease is caused by the *Mycobacterium tuberculosis* (MTB) complex. TB typically invades the lungs and affects other organs of the human body (extrapulmonary TB). Lymph nodes are the most common site of extrapulmonary TB [1]; intrathoracic lymph nodes, including mediastinal and hilar lymph nodes, are particularly affected, resulting in TB intrathoracic lymphadenopathy (TBIL). In adults, TBIL may be an isolated finding or associated with lung infiltration.

Sarcoidosis (SA) is a multisystem autoimmune disease, which primarily affects the lungs. The most common extrapulmonary organs involved in this disease are the skin, lymph nodes (LN), eyes, and liver [2]. Sarcoidosis intrathoracic lymphadenopathy (SAIL), including Scadding stages I and II, is the most common form of sarcoidosis intrathoracic lymphadenopathy. The etiology of sarcoidosis is currently unknown. *MTB* was previously considered to be the cause of sarcoidosis. However, recent studies have suggested that mycobacterial antigens are not the pathogenic cause of sarcoidosis [3, 4]. Therefore, finding the evidence of TB infection is the key for distinguishing these two diseases. Owing to their similar clinical and radiological profiles, as well as the same granulomatous pathological features when there was no caseous necrosis, it is difficult to distinguish sarcoidosis from TB. Moreover, extrapulmonary TB samples carry a lower *MTB* load than respiratory samples. This difference affects the sensitivity of conventional methods in diagnosing TB, including smear microscopy, culture, or cytology.

Mycobacterial culture remains the gold standard for the diagnosis of active TB. However, this approach requires 2–4 weeks to yield results, and its sensitivity for lymphatic TB is poor [5]. In 2013, the World Health Organization recommended the use of Xpert MTB/resistance to rifampin (Xpert MTB/RIF, referred to as Xpert) for some types of extrapulmonary TB, such as TB lymphadenitis and TB meningitis [6]. The cost of this method is similar to that of *MTB* culture; however, it offers the advantages of a short cycle, faster process, automation. A meta-analysis analyzed 18 studies involving 4,461 samples; they found that, in lymph node tissues or aspirates, the pooled sensitivity of Xpert versus culture was 83.1% (95% confidence interval: 71.4–90.7%) [7]. Moreover, it can be used for differentiating tuberculosis from sarcoidosis [8].

In the present study, we aimed to evaluate the role of Xpert in the detection of *MTB* for differentiating TBIL from SAIL. To achieve our objective, we used fresh specimens obtained through endobronchial ultrasound-guided transbronchial needle aspiration (EBUS-TBNA, referred to as EBUS). Moreover, we compared the clinical features of the two diseases to detect differences that could assist us in reaching a definitive diagnosis.

## Methods

### Study design

All patients (n = 119) suspected to having SAIL or TBIL during the initial inpatient period at Shanghai Pulmonary Hospital (Shanghai, China) between October 1, 2020 and June 30, 2021 were retrospectively evaluated and followed-up for > 6 months. The last follow-up was performed on February 1, 2022. All patients underwent EBUS-TBNA from the mediastinal, hilar, and interlobar LN to obtain tissue specimens for Xpert analysis.

### Inclusion and exclusion criteria

The inclusion criteria were: (1) age 18–80 years; (2) suspected SAIL or TBIL with or without lung infiltration during the initial inpatient period diagnosed by two specialist physicians and two radiologists; (3) final diagnosis of sarcoidosis and TB during the follow-up period; (4) having underwent EBUS-TBNA and Xpert examinations; (5) negative sputum acid-fast bacilli (AFB) smear prior to EBUS; and 5) no treatment with anti-TB drugs, glucocorticoids, or immunosuppressive agents for > 1 month prior to EBUS.

The exclusion criteria were: (1) presence with any malignancy; (2) presence of fungal or other infections detected through histopathological or microbiological analysis; (3) lack of a correct definitive diagnosis during the follow-up period; and (4) lost to follow-up.

The medical records of all patients, including demographic data (sex, age, and race), medical history (symptoms, presence of comorbidities, diagnostic test, and treatment outcome), laboratory results, and the features of chest high-resolution computed tomography (HRCT) were collected.

### Endobronchial ultrasound-guided transbronchial needle aspiration and ultrasonography procedure

EBUS-TBNA was performed using a dedicated bronchoscope with a linear ultrasound transducer (BF-UC260F-OL8; Olympus, Tokyo, Japan). The target LN were punctured with a 21-gauge needle (NA-201SX-4022; Olympus, Tokyo, Japan). All patients provided written informed consent before undergoing the bronchoscopy. Two or more punctures were performed on each target lymph node to obtain at least two tissue core specimens. One of those specimens was prepared for histological examination, while the other specimens were utilized for AFB, Xpert, and MGIT960 culture. Ultrasonography was conducted with model HDI 5000, 7–12 MHz (Philips Medical Systems, Bothell, WA, USA) to evaluate the sizes (in cm) of superficial LN by measuring the largest diameters. Nodal masses with size > 5 mm were identified as bulky lesions. The size of LN was assessed by measuring the largest and smallest diameters on the ultrasound screen, and the long axis/short axis (L/S) ratio was calculated.

### Xpert MTB/RIF procedure

The rpoB gene is the target of Xpert. The Xpert assay was performed according to the instructions provided by the manufacturer (Cepheid, Sunnyvale, CA, USA). The sample reagent was added to ≥ 0.5 mL of decontaminated specimen at a 3:1 ratio. After shaking, the mixture was incubated for 15 min at room temperature. Thereafter, part of the mixture (2 mL) was transferred to the Xpert test cartridge. The semiquantitative results of the Xpert were obtained based on the cycle threshold in each sample using the Xpert software within 2 h.

### Diagnosis of TBIL and SAIL

#### TBIL

The diagnosis of TBIL was based on the presence of *MTB* in all samples. Biopsies performed using pathological tissues showed granulomatous reaction with caseation necrosis or multinucleated giant cells associated with epithelioid histiocytes. Possible (based on clinical, imaging, and histological assessments in this study) TB according to the European Centre for Disease Prevention and Control [9] when the symptoms improved, the size of LN was decreased, and/or the lung infiltration resolved after the standard anti-TB treatment is also considered as TBIL.

#### SAIL

The diagnosis of mediastinal sarcoidosis was reached according to the official American thoracic society clinical practice guideline [10]. Histological specimens showed non-caseous necrotizing granulomas without other known causes of granulomatosis (evidence of TB, fungi, etc.). Stage I (mediastinal or hilar lymphadenopathy) and stage II (lymphadenopathy accompanied by pulmonary infiltrations) were included.

### Statistical analysis

The mean ± standard deviation was used for measurement data, while data with skewed distribution were described using the interquartile range. For continuous variables, the *t*-test was used to determine mean differences between groups. Non-normally distributed data were analyzed by the Mann–Whitney *U* test. For categorical variables, differences were assessed using the chi-squared or Fisher’s exact test. Two-tailed P-values < 0.05 indicate statistically significant differences. The sensitivity, specificity, positive predictive value (PPV), and negative predictive value (NPV) of various diagnostic methods were calculated. All statistical analyses were performed using IBM SPSS statistics version 23.0 (IBM Corp., Armonk, NY, USA).

## Results

### Patient characteristics

**Fig. 1:**
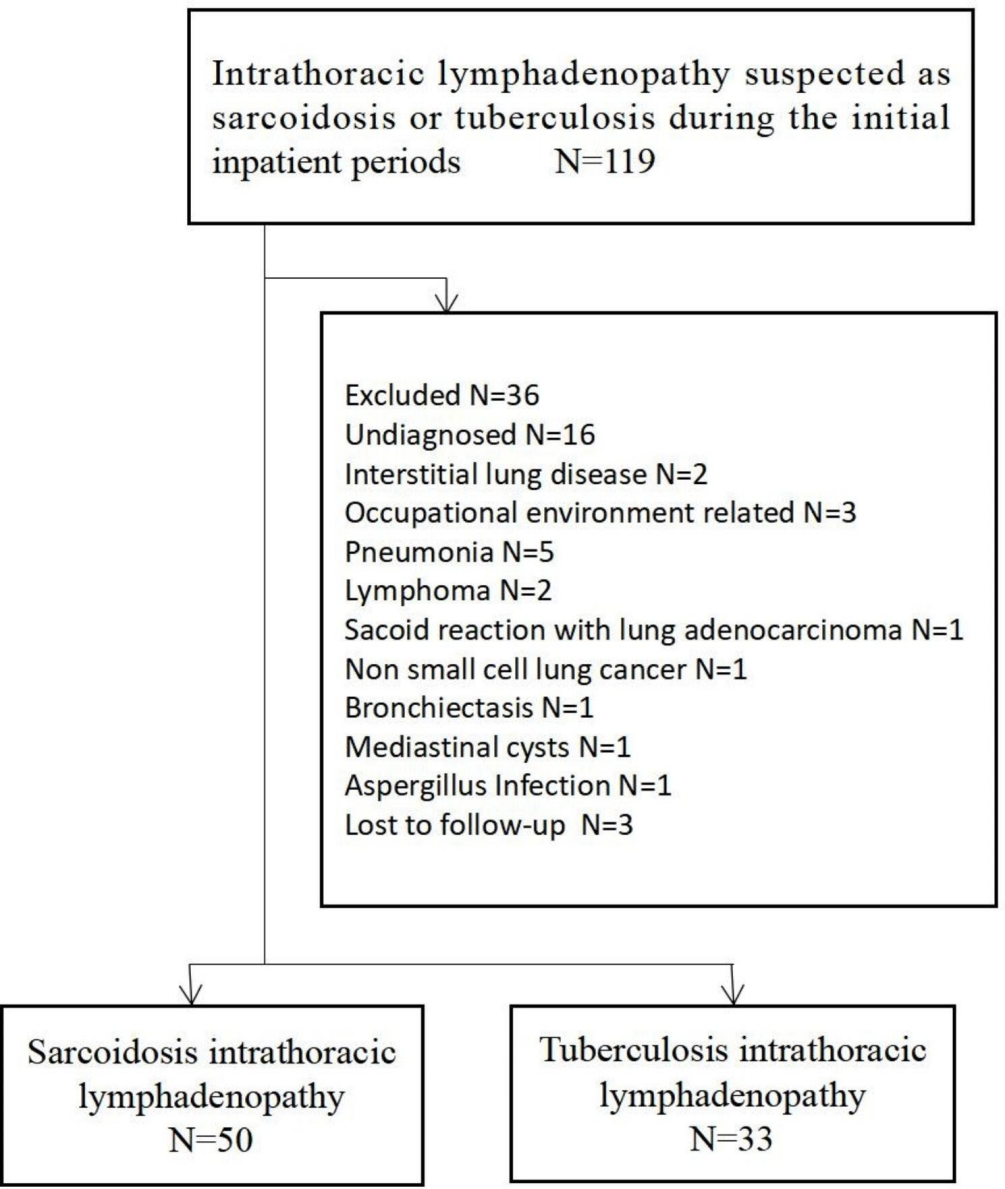
Flow chart of intrathoracic lymphadenopathy

Of the 119 patients analyzed, 83 were eventually diagnosed with SAIL (N = 50) and TBIL (N = 33) (Fig. 1). All patients underwent EBUS-TBNA and Xpert analysis. We found that young individuals were more likely to have TBIL than SAIL (Table 1).

**Table 1:** Baseline characteristics of study patients

	SAIL	N	TBIL	N	P-value
Age, years	53 (42.5–58)	50	34 (26.5–54.5)	33	0.006
Female/Male, N	38/12	50	16/17	33	0.01
BMI, kg/m^2^	24.35 ± 3.38	50	22.48 ± 2.40	33	0.007
Serum amyloid A, mg/L	8.32 (5.95–11.66)	45	9.845 (5.5975–33.225)	30	0.168
Hemoglobin, g/L	131.12 ± 15.33	50	125.33 ± 18.19	33	0.122
Leukocytes, ×10^9^/L	4.79 ± 1.25	50	6.22 ± 2.12	33	< 0.01
Neutrophils%	62.72 ± 7.15	50	63.18 ± 10.02	33	0.804
Eosinophils%	2.4 (1.675–4.1)	50	1.5 (0.85–2.55)	33	0.001
Lymphocytes%	22.40 ± 7.33	50	24.78 ± 8.78	33	0.183
Monocytes%	11.42 ± 5.14	50	9.78 ± 3.25	33	0.107
Albumin, g/L	41 (38–44)	50	40 (38–42)	33	0.343
Globulin, g/L	25.5 (23.75–30.25)	50	27 (23–29.5)	33	0.797
Erythrocyte sedimentation rate, mm/L	23.5 (12.25–44.5)	48	29 (12–69.75)	28	0.194
C-reactive protein, mg/L	3.3 (3.1–4.3)	47	4.7 (3.1–27.4)	19	0.100
Serum ferritin	97.18 ± 58.66	46	158.54 ± 110.48	24	0.003
Serum calcium	2.29 (2.205–2.41)	50	2.34 (2.22–2.4325)	33	0.399
IgG, g/L	13.08 ± 2.61	46	13.97 ± 3.87	15	0.313
IgA, g/L	2.345 (1.95–3.195)	46	2.53 (2.16–3.37)	15	0.311
IgM, g/L	0.93 (0.66–1.265)	46	0.84 (0.65–1.32)	15	0.633
IgE, IU/mL	26.95 (17.8–51.95)	48	58.55 (17.8–148.25)	20	0.095
C3, g/L	1.26 ± 0.19	46	1.32 ± 0.27	15	0.305
C4, g/L	0.25 ± 0.70	46	0.27 ± 0.09	15	0.485
CD4	40.93 ± 9.26	35	37.43 ± 8.15	21	0.159
CD8	19.32 ± 9.86	35	23.36 ± 7.33	21	0.110
CD4/CD8	2.2 (1.41–3.27)	35	1.75 (1.21–2.005)	21	0.020
24-h urinary calcium	5.98 (4.44–8.00)	43	2.75 (1.98–10.09)	5	/
Serum angiotensin, IU/L	43 (27–66.25)	50	21 (16–46)	19	0.003

BMI, body mass index; SAIL, sarcoidosis intrathoracic lymphadenopathy; TBIL, tuberculosis intrathoracic lymphadenopathy

In addition, a lower proportion of women had TBIL compared with SAIL (N = 16, 48.48% vs. N = 38, 76%; P = 0.01). The body mass index was lower in patients with TBIL versus SAIL. Chest distress (P = 0.001) and dyspnea (P = 0.010) more often occurred in patients with SAIL, while fever (P = 0.002) was more frequently observed in those with TBIL. There were no differences detected in other symptoms (Supplementary Table 1). Markers of inflammation, including serum amyloid A, leukocytes, erythrocyte sedimentation rate, C-reactive protein levels, and serum ferritin concentration, were higher in TBIL versus SAIL, particularly leukocytes and serum ferritin (P < 0.01). Although the eosinophil ratio was significant higher in SAIL versus TBIL, only seven patients exhibited values above the normal. The patients with SAIL had higher serum angiotensin levels than those with TBIL. Extrathoracic involvement was more often observed in SAIL compared with TBIL (29 vs. 9, respectively; P = 0.006) (Supplementary Table 1). There were no significant differences in comorbidities (emphysema, coronary heart disease, hypertension, and diabetes) between SAIL and TBIL (P = 0.283) (Supplementary Table 1).

### Imaging features

We found that most patients with intrathoracic lymphadenopathy had concurrent pulmonary infiltration. Notably, 92% (46/50) of SAIL patients and 87.88% (29/33) TBIL patients exhibited lung involvement (P = 1.000). Moreover, patients with SAIL were at a higher risk of infiltration into bilateral lungs versus those with TBIL (90.91% vs. 37.93%, respectively; P < 0.01). The largest diameter of lung lesions was smaller in patients with SAIL versus TBIL (P < 0.01) (Table 2).

**Fig. 2:**
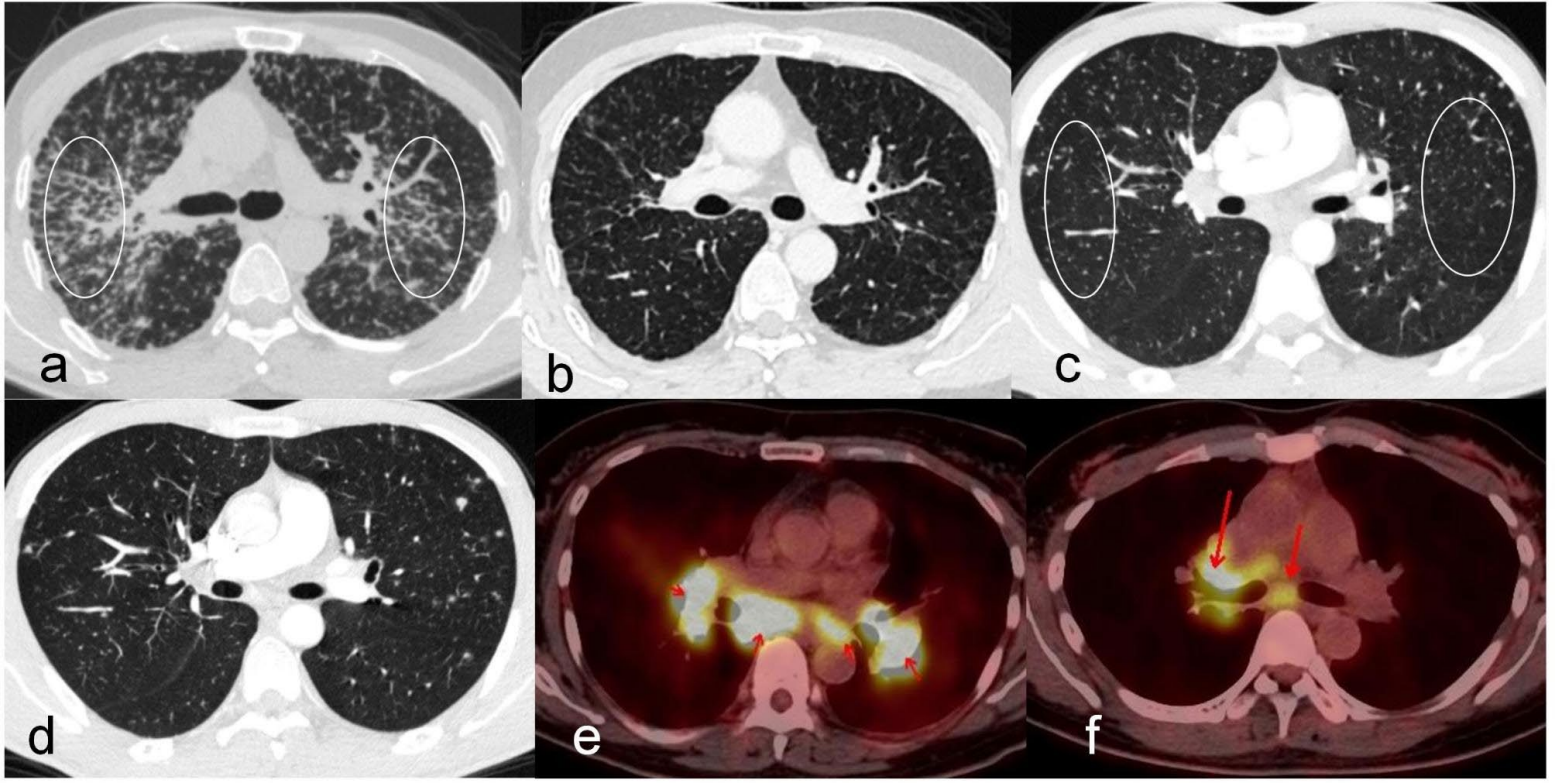
HRCT and PETCT features of SAIL and TBIL. (**a** and **b**) HRCT showing miliary nodules in both the bronchovascular bundle and interlobular septa before (**a**) and 6 months after (**b**) treatment of a male patient with prednisone. (**c** and **d**) HRCT showing that most miliary nodules were similar in terms of size, and their distribution bears no relation to the airways before (**c**) and 12 months after (**d**) treatment of a male patient with anti-TB therapy. (e and f) PETCT indicating bilateral and symmetrical of SAIL (**e**) and asymmetric unilateral TBIL (**f**) HRCT, high-resolution computed tomography; PETCT, positron emission tomography–computed tomography; SAIL, sarcoidosis intrathoracic lymphadenopathy; TB, tuberculosis; TBIL, tuberculosis intrathoracic lymphadenopathy

A significantly larger number of stations of intrathoracic lymphadenopathy (e.g., 2R/L, 4R/L, 5, 6, 7, 8, 9, 10R/L, and 11R/L) involved per patient were detected in SAIL versus TBIL (P < 0.01). Similarly, the LN were more likely to show symmetry in patients with SAIL versus TBIL (P < 0.01). TBIL is mostly asymmetric, and the largest involved sites were the right paratracheal (2R [N = 6] and 4R [N = 7]) and subcarinal lymph (station 7 [N = 14]) (Fig. 2). Station 7 (N = 46) was the largest site involved in SAIL (P < 0.01). The longest diameter of intrathoracic lymphadenopathy (measured in cm) of SAIL was significantly larger than that of TBIL. Nonetheless, patients with TBIL had significantly higher lymph node calcification than those with SAIL. Stations 4R and 7 were the most common sites of lymph node puncture in these two diseases.

Eleven and six patients with SAIL and TBIL, respectively, underwent positron emission tomography–computed tomography (PETCT). Among patients with SAIL, seven (63.64%), two, and two were diagnosed with sarcoidosis, infection disease, and malignant tumor, respectively. Among patients with TBIL, two and four patients were diagnosed with TB and malignant tumor, respectively.

**Table 2:** Lung and lymph nodes features of HRCT and ultrasonography

	SAIL, N	TBIL, N	P-value
Lung involvement			
None	6	4	1.000
Bilateral	40	11	<0.01
Unilateral	4 (left/right, 1/3)	18 (left/right, 5/13)	
Longest diameter of lung lesions (cm)	0.98 (0.7925–1.4475)	2.05 (0.86–2.875)	0.005
Major radiology features			
Nodules	37	20	0.126
Consolidation	4	6	
Others	3	3	
Numbers of IL involved per patient	12 (12–12)	3 (2–4)	< 0.01
Symmetry of MLN	47/3	8/25	< 0.01
Longest diameter of MLN (cm)	4.02 (3.15–4.9425)	3.12 (2.68–3.685)	0.001
Stations of LN puncture			
2R	1	5	
4R	42	21	
4 L	7	7	
7	45	25	
10R	0	0	
10L	0	1	
11R	13	8	
11L	12	2	
Intrathoracic LN calcification	6/44	11/22	0.018
Superficial lymphadenopathy (Yes/No)	35/7	21/10	0.119
Longest diameter of superficial LN (cm)	13 (11.5–17.7)	14.6 (10.3–20.65)	0.741
L/S ratio	1.74 ± 0.36	1.69 ± 0.56	0.667
Supraclavicular/others	26/9	16/5	0.873
Left/Right	14/21	4/17	0.104
Bronchial involvement	7/43	9/24	0.134

HRCT, high-resolution computed tomography; LN, lymph nodes; L/S, long axis/short axis; MLN, mediastinum lymph nodes; SAIL, sarcoidosis intrathoracic lymphadenopathy; TBIL, tuberculosis intrathoracic lymphadenopathy

### Involvement of other organs

The rate of extrathoracic organ involvement (particularly skin, eyes, spleen, and kidneys) was higher in SAIL versus TBIL (Supplementary Table 1). The supraclavicular LN were the mostly frequently involved site compared with cervical, axillary, and inguinal LN. Necrosis of superficial lymph node tissues was more commonly noted in TBIL versus SAIL (N = 6, 42.86% vs. N = 1, 5.26%, respectively; P = 0.026). There were no significant differences in the longest diameters and L/S ratios between SAIL and TBIL. Endobronchial nodules and bronchial pachymucosa/mucosal swelling (nodules [N = 4] and others [N = 3]) were major features of bronchial involvement in SAIL. Neoplasm with caseous necrosis, edematous-hyperemia, ulcer, and tracheobronchial stenosis were often observed in TBIL (neoplasm with caseous necrosis [N = 5] and others [N = 4]).

### Diagnostic performances of Xpert, AFB, culture, and QuantiFERON-TB gold

Xpert did not detect *MTB* in all patients with SAIL. Among those with TBIL, positive results were obtained for 23 (69.70%) patients (P < 0.01). This approach yielded negative findings for other diseases and undiagnosed diseases (Table 3).

**Table 3:** Comparison of different methods in differentiating sarcoidosis from tuberculosis intrathoracic lymphadenopathy

	EBUS Xpert		EBUS AFB		EBUS culture		QFT		*Combined	
	positive	negative	positive	negative	positive	negative	positive	negative	positive	negative
SAIL, N	0	50	0	50	0	50	4	45	4	46
TBIL, N	23	10	1	32	8	25	27	1	31	2
Sensitivity	69.70%		3.03%		24.24%		96.43%		93.94%	
Specificity	100%		100%		100%		91.84%		92%	
PPV	100%		100%		100%		87.10%		88.57%	
NPV	83.33%		60.98%		66.67%		97.83%		95.83%	
Accuracy rates	87.95%		61.45%		69.88%		93.51%		92.77%	
P-value	< 0.01		0.216		< 0.01		< 0.01		< 0.01	
Other diseases, N	0	17	0	17	0	17	8	9	8	9
Undiagnosed diseases, N	0	16	0	16	0	16	9	6	9	6

*The results of combined EBUS Xpert and QFT (positive results from either EBUS Xpert or QFT denoted TB)

AFB, acid-fast bacilli; EBUS, the short form of endobronchial ultrasound-guided transbronchial needle aspiration; NPV, negative predictive value; PPV, positive predictive value; QFT, QuantiFERON-TB Gold; SAIL, sarcoidosis intrathoracic lymphadenopathy; TB, tuberculosis; TBIL, tuberculosis intrathoracic lymphadenopathy

Xpert yielded negative results for 10 patients with TBIL. The diagnosis of these 10 patients was reached as follows: diagnosed by the histopathologic findings with caseous necrosis of cervical lymph nodes (N = 2); concurrent with endobronchial TB (N = 1); associated with lung TB diagnosed by positive bronchoalveolar lavage fluid Xpert (N = 1); diagnosed by positive sputum TB culture (N = 2); and 4 patients (two with positive AFB of bronchoscopy brush specimens) associated with lung involvement were confirmed to having TB by the alleviated clinical symptoms and radiological features after 6 months of anti-TB therapy. The rate of positive results from the culture of samples obtained through EBUS-TBNA were 0% and 24.24% in SAIL and TBIL, respectively (P < 0.01). There was no significant difference in EBUS AFB between the two diseases. QFT examination of peripheral blood yielded positive results for 27 (96.43%) and four (8.16%) patients with TBIL and SAIL, respectively. The results were inconclusive for two patients with TBIL and one patient with SAIL. Three patients with TBIL did not undergo QFT.

## Discussion

TB lymphadenitis is more frequent in children and females, with the peak age of onset ranging 15–30 years in high TB burden countries [11]. In the present study, the age of patients with TBIL ranged 26.5–54.5 years and only 36.36% (12/33) were aged > 40 years, these patients were markedly younger than those with SAIL (80%, 40/50, p < 0.01). TB is an infectious disease caused by *MTB*. Although most patients with TB lymphadenitis did not exhibit special symptoms [12], those with TBIL were more likely to experience fever in our study. Moreover, indicators of infection (i.e., leukocytes, erythrocyte sedimentation rate, C-reactive protein, serum amyloid A, and serum ferritin) were higher in TBIL, particularly leukocytes and serum ferritin. These data suggested that, unlike SAIL, TBIL is an infectious disease. Importantly, 30–60% of patients with sarcoidosis are asymptomatic, and the disease is often discovered accidentally during a chest examination. In this study, patients with SAIL were more likely to experience chest distress and dyspnea versus those with TBIL, which may be related to the compression caused by mediastinal lymph node enlargement. Renston et al. found that 41% of patients with sarcoidosis had peripheral blood eosinophilia [13]. We found that the ratio of eosinophils in peripheral blood was significantly higher in SAIL versus TBIL, which may indicate different pathogenic processes. Nevertheless, further studies are required to determine how eosinophils participate in the pathogenesis of sarcoidosis. In addition, there were no differences in the nutritional and immune status of patients in the two groups in terms of albumin, globulin, IgG, IgM, IgA, IgE, C3, C4, CD4, and CD8 levels. However, patients with SAIL had a higher body mass index than those with TBIL (Table 1).

In intrathoracic sarcoidosis, the rate of bilateral hilar lymphadenopathy is 20–65%, while that of bilateral hilar lymphadenopathy associated with pulmonary infiltration is 20–40% [14]. Lymph nodes, especially thoracic LN, are among the most common sites of extrapulmonary TB [15]. However, the presence of isolated TBIL without a parenchymal lung lesion in adults is unusual, with an incidence rate of 0.25–5.8% [16]. In this study, solely mediastinal lymph node enlargement was not common (12% and 12.12% for SAIL and TBIL, respectively). Nodules were the main pulmonary features of the two diseases, including single, discrete, and miliary patterns. The formation of sarcoidosis granulomas occurs nearly exclusively along the lymphatic tracks. Hence, the typical feature of pulmonary sarcoidosis was nodular involvement in both the bronchovascular bundle and interlobular septa. This feature was typically symmetrical and demonstrated upper and middle lung zone predominance (Fig. 2). Miliary TB usually results from the acute hematogenous dissemination of TB bacilli in lungs. These nodules are uniform in size and their distribution bears no relation to the airways [17] (Fig. 2). There were no cavities in the lungs of patients with these two diseases. On computed tomography, LN in sarcoidosis are usually discrete, bilateral, and symmetrical, and rarely show a central hypodensity. In contrast, central necrosis and asymmetric conglomerate LN are frequently observed in TB [18]. Consistently, in our research, patients with TBIL mainly had asymmetric unilateral LN, especially in the drainage area of pulmonary lesions. Calcifications of the mediastinal or hilar LN were frequently observed in TBIL. In this study, extrathoracic organ involvement was more easily noted in SAIL versus TBIL.

It is critical to find evidence of *MTB* infection for differentiating sarcoidosis and TB. A positive culture for *MTB* using different specimens is currently the gold standard for the diagnosis of TB. However, the rate of positive culture was low for cases of lymphatic TB (2.1%) [19] due to difficulty in sampling from the LN and the paucibacillary nature of the specimens. Therefore, a rapid laboratory test is necessary for the diagnosis of TB. Xpert is an automated diagnostic test for the detection of *MTB*. It is a DNA-based test that detects the *MTB* rpoB gene [20]. The extraction, amplification, and detection by Xpert take place within a single-use multichambered cartridge, thus minimizing the risk of sample contamination. This method can provide results within 2 h. In this study, we used Xpert to find evidence of TBIL using specimens obtained through EBUS-TBNA. The rates of sensitivity, specificity, PPV, NPV, and accuracy of Xpert for differentiating SAIL from TBIL were 69.70%, 100%, 100%, 83.33%, and 87.95% respectively; these rates were higher than those of EBUS AFB and EBUS culture (Table 3). EBUS Xpert exhibited a higher PPV, suggesting that positive results in EBUS Xpert were associated with a higher likelihood for diagnosis of TB.

At present, the available tests are unable to accurately predict the progression of latent TB infection to active TB. In a systematic review, it was found that the interferon-gamma (IFN-γ) release assays (IGRAs) have a higher PPV than tuberculin skin tests. These findings suggested that individuals with positive IGRA results are more likely to progress to active TB than those with negative results [21]. QFT is a type of IGRA. Piotrowski proposed the use of QFT as a cost-effective diagnostic test, which can provide additional diagnostic information when a false-positive MTB culture result in a patient with sarcoidosis is highly suspected [22]. In the present study, the rates of sensitivity, specificity, PPV, NPV, and accuracy of QFT for differentiating SAIL from TBIL were 96.43%, 91.84%, 87.10%, 97.83%, and 93.51% respectively. These rates were higher than those of EBUS Xpert, except for specificity and NPV because there were also positive results in other and undiagnosed diseases. QFT with a highest NPV suggested that individuals with a negative QFT were more likely to exclude the diagnosis of TB and the lowest PPV indicated that if the patient had a positive QFT was not so strongly recommended to have the diagnosis of TB. Therefore, although QFT cannot provide a definitive diagnosis of active TB, it can assist us in excluding TB.

### Limitations

First, owing to the insufficient number of specimens, some patients had false-negative results on EBUS Xpert, thereby decreasing the sensitivity of the method. Second, the QFT test was not performed in all patients, which may reduce the credibility of the results. Finally, this was a single-center retrospective study; hence, a multi-center prospective study is warranted to confirm the present findings.

## Conclusions

Patients with TBIL were more likely to have an infection than those with SAIL; however, patients with SAIL were at a higher risk of extrathoracic involvement. Intrathoracic lymph nodes in SAIL were typically bilateral, symmetrical, and multifocal. Furthermore, the pulmonary radiological features of SAIL were bilateral nodes in both the bronchovascular bundle and interlobular septa. The use of EBUS Xpert is recommended for the diagnosis of TBIL. A negative QFT result can be utilized to exclude the diagnosis of TBIL.

### Electronic supplementary material

Below is the link to the electronic supplementary material.

Supplementary Material 1

## Data Availability

All data generated or analyzed during this study are included in this published article and supplementary materials.
