# Supplementary material for: Xpert MTB/RIF assay for the differential diagnosis between sarcoidosis and tuberculosis intrathoracic lymphadenopathy
Source: BMC Infect Dis. 2023 Oct 25;23:725. doi: 10.1186/s12879-023-08734-7 (PMC10601222; doi:10.1186/s12879-023-08734-7)
Supplement: Supplementary file 1 — Supplementary Material 1 [file 12879_2023_8734_MOESM1_ESM.docx]

**Supplementary Table 1** Clinical manifestation and comorbidities of SAIL and TBIL

|  | SAIL, n | TBIL, n | *p* |
| --- | --- | --- | --- |
| Symptoms | | | |
| Body exam | 15 | 6 | 0.225 |
| Cough | 25 | 21 | 0.221 |
| Sputum | 14 | 10 | 0.821 |
| Chest distress | 19 | 2 | 0.001 |
| Dyspnea | 12 | 1 | 0.010 |
| Fatigue | 4 | 1 | 0.646 |
| Weight loss | 4 | 2 | 1.000 |
| Night sweat | 3 | 1 | 1.000 |
| Hemoptysis | 1 | 4 | 0.079 |
| Fever | 1 | 9 | 0.002 |
| Chest pain | 8 | 3 | 0.563 |
| Extrathoracic involvements | | | 0.006 |
| Skin | 5 | 0 |  |
| Eye | 8 | 0 |  |
| Pericardium | 3 | 2 |  |
| pleura | 7 | 5 |  |
| Liver | 0 | 0 |  |
| Spleen | 3 | 1 |  |
| kidney | 3 | 0 |  |
| Nervous system | 0 | 1 |  |
| Accompanied with | | | 0.283 |
| Emphysema | 2 | 1 |  |
| Coronary heart disease | 2 | 1 |  |
| Hypertension | 7 | 4 |  |
| Diabetes | 5 | 1 |  |
